# Analysis of Byzantine Attacks for Target Tracking in Wireless Sensor Networks

**DOI:** 10.3390/s19153436

**Published:** 2019-08-05

**Authors:** Yukun Yang, Pengwen Xiong, Qing Wang, Qiang Zhang

**Affiliations:** School of Information Engineering, Nanchang University, Xuefu Road No. 999, Honggutan New District, Nanchang 330031, China

**Keywords:** wireless sensor networks, target tracking, Byzantine attacks

## Abstract

Herein, the problem of target tracking in wireless sensor networks (WSNs) is investigated in the presence of Byzantine attacks. More specifically, we analyze the impact of Byzantine attacks on the performance of a tracking system. First, under the condition of jointly estimating the target state and the attack parameters, the posterior Cramer–Rao lower bound (PCRLB) is calculated. Then, from the perspective of attackers, we define the optimal Byzantine attack and theoretically find a way to achieve such an attack with minimal cost. When the attacked nodes are correctly identified by the fusion center (FC), we further define the suboptimal Byzantine attack and also find a way to realize such an attack. Finally, in order to alleviate the negative impact of attackers on the system performance, a modified sampling importance resampling (SIR) filter is proposed. Simulation results show that the tracking results of the modified SIR filter can be close to the true trajectory of the moving target. In addition, when the quantization level increases, both the security performance and the estimation performance of the tracking system are improved.

## 1. Introduction

Wireless sensor networks can be flexibly deployed in various application environments and perform tasks such as the sensing, acquisition, processing, and transmission of target information. When the perceived information needs to combine with nodes’ locations to develop its own value, the self-localization process of sensor nodes becomes the application premise of wireless sensor networks (WSNs). In practical applications, when WSNs are deployed in a non-secure environment, the sensor nodes may be subjected to various attacks. Through modifying the reference data (such as anchor positions or ranging information), the attackers can produce severe damage to the localization accuracy [1,2,3,4,5].

How to prevent attackers from modifying the reference data or how to realize reliable localization under attack has always been the research focus in the field of secure localization. In the past decades, researchers have proposed many reliable localization strategies. The most intuitive strategy is to employ some techniques to protect the integrity of the reference data and make the observation process robust. This strategy can be called the secure localization strategy based on robust observations. The representative work includes the distance bounding protocol [6] and the SeRLoc algorithm [7]. These methods mainly use the time constraints, space constraints, or signal coding techniques to protect the physical properties of beacon information. However, this type of method relies on additional hardware units and is not suitable for large-scale promotion.

When modified observations from attackers (or as we called them, the malicious observations) are unavoidable, the researchers propose detecting and eliminating the malicious observations and then using the remaining honest observations to achieve node localization [8,9,10]. This strategy can be called the secure localization strategy based on malicious node detection. A typical work is the MEF-based localization algorithm proposed in Reference [8]. A common feature of this type of method is that the detection of malicious nodes usually needs to compare a large amount of data, thus causing a heavy calculation overhead. Meanwhile, a certain type of detection method can only detect a specific type of malicious attack. So, the applicability of this kind of method is weak.

In order to reduce the requirements on the hardware, and also to improve the applicability of secure localization algorithms, some researchers choose to develop methods to improve the robustness of the position computation process (i.e., the key process of node localization). In the traditional trilateration method, the position estimates are derived in the sense of least squares. Since the cost function of this method is the sum of the squared errors of all sample data, it is very sensitive to the outliers. A single malicious observation may cause a serious deviation in the position estimate. In response to this problem, Li et al. [11], proposed a positioning mechanism based on the idea of least median of squares, which estimates the unknown parameters by minimizing the median of the residual squares. Results show that, in the absence of measurement noise, even if there are 50% of outliers in the observation data, this method can still output the correct position estimate. In Reference [12], the authors combined iterative gradient descent with selective pruning of inconsistent measurements to achieve reliable localization. During each iteration, the forward direction is corrected by eliminating the suspicious gradient vectors, thereby ensuring that the iterative path is constantly approaching the true position of the unknown node. A common feature of the above methods is that they enhance the reliability of the positioning system by improving the robustness of the position computation process. Therefore, this type of strategy can be called the secure localization strategy based on robust computing.

Most existing secure localization algorithms study how to defend against malicious attacks from the perspective of defenders. Few articles examine the impact of different attack strategies on the positioning systems from the perspective of attackers. This paper focuses on the target tracking problem under Byzantine attacks and investigates the optimal Byzantine attack strategy for malicious nodes in different situations. The prototype of Byzantine attacks comes from the issue of Byzantine generals [13], where some traitors try to confuse other loyal generals by delivering false information. Here, we apply the malicious behaviors of delivering false data into sensor networks. For the fragile sensor networks, such a type of attack is easy to implement. A typical example is the man-in-the-middle (MiM) attack [14]. In the MiM attacks, the attacker first disguises itself as a legal fusion center (FC) to collect data from sensor nodes. Then, they modify the data and send false information to the real FC.

Vempaty et al. [15,16], analyzed the distributed estimation problem under Byzantine attacks. In their model, the attackers are unaware of the true states of the target, the quantization scheme employed by each node, and the estimation method used by the FC. They can only access and modify the quantized results of attacked nodes. By means of the posterior Cramer–Rao lower bound (PCRLB), the authors successfully quantized the impact of a Byzantine attack on the system performance and derived the minimum number of attacked nodes to achieve the maximum degradation to the system performance. Based on the above, Nadendla et al. [17], extended the system framework from binary quantization to *L*-dimensional (*L* ≥ 2) quantization and found the optimal Byzantine attack that blinds any distributed inference network. In Reference [18], the authors investigated the optimal processing of honest observations and malicious observations. When the number of observations or the total number of nodes approaches infinity, the authors theoretically proved that the FC has the ability to classify all nodes according to the types of attacks.

**Figure 1:**
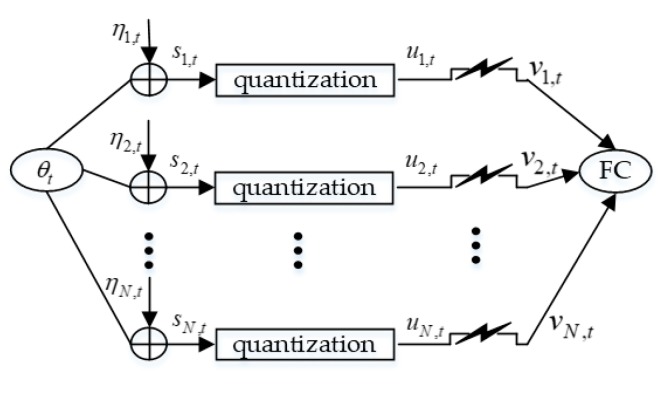
Simplified system model. The value $\eta_{i,t}$ is measurement noise of the *i*th sensor, $s_{i,t}$ is the raw measurement, $u_{i,t}$ is the quantized sensor measurement, and $v_{i,t}$ is the measurement received by the fusion center, where *i* = 1, …, *N*.

In this paper, we consider a WSN that is deployed for the purpose of tracking the real-time state (denoted as $\theta_{t}$) of a moving target. After obtaining noisy measurements about the target, the sensors first quantize their raw observations and then send the quantized measurements to a fusion center, which is responsible for estimating $\theta_{t}$. Figure 1 shows the simplified model of the entire system. Here, we extend the framework of the target tracking problem in Reference [19] to a more general case where sensors use *L*-dimensional quantization schemes. The PCRLB for total unknowns (including unknown target states and unknown attack parameters) is calculated. From the perspective of attackers, we define the optimal Byzantine attack and derive how to achieve such an attack with a minimal cost. When all attacked nodes are correctly identified by the FC, we further define the suboptimal Byzantine attack and also find a way to realize such an attack. In order to alleviate the negative impact of Byzantine attacks, we propose a modified SIR filter. Simulation results show that by using the modified SIR filter, the tracking results can be very close to the true trajectory of the moving target. In addition, when the quantization level increases, the security performance and the estimation performance of the tracking system are both improved.

The remainder of this paper is organized as follows. Section 2 describes the system model for the target tracking problem under Byzantine attacks. Next, we calculate the PCRLB of the unknowns and determine the optimal and suboptimal attack strategy for the attackers in Section 3. In Section 4, the modified SIR filter is proposed, and simulation results are also presented. Finally, conclusions are made in Section 5.

## 2. System Model

We consider a single target moving in a 2-dimensional plane whose dynamics is defined by a state vector, $\theta_{t}={\left[x_{t},y_{t},vx_{t},vy_{t}\right]}^{T}$, where $x_{t}$ and $y_{t}$ are the *x* and *y* coordinates of the moving target in the time unit, *t*, respectively. The values $vx_{t}$ and $vy_{t}$ denote the velocities in the *x* and *y* directions. The evolution of the target state sequence is defined as follows:

(1) $$\theta_{t}=\tilde{F}\theta_{t-1}+w_{t},$$

where $\tilde{F}$ is the state transition matrix and $w_{t}$ is the process noise, which is assumed to be white and Gaussian with zero mean and covariance matrix, $Q_{1}$. It is assumed that the FC has exact knowledge of the target state-space model and the process noise statistics.

In order to track the real target state, a sensor network consisting of *N* spatially distributed sensors is deployed. Each sensor measures the signal emitted from the target. The measured signal at each sensor follows:

(2) $$\left\{ \begin{array}{l} s_{i,t}=a_{i,t}+\eta_{i,t}\\ a_{i,t}^{2}=P_{0}{\left(d_{0}/d_{i,t}\right)}^{\alpha } \end{array}\right.,$$

where $s_{i,t}$ is the received signal amplitude at the *i*th sensor at time instant *t*. The measurement noises, ${\left\{\eta_{i,t}\right\}}_{i=1}^{N}$, are assumed to be independent across sensors and follow a common Gaussian distribution, $N(0,\sigma^{2})$. The value *P*_0_ is the measured power at the reference distance *d*_0_, α is the path-loss exponent, and $d_{i,t}$ is the distance between the target and the *i*th sensor. Without loss of generality, we assume *d*_0_ = 1 and α = 2.

Due to the energy and bandwidth constraints, each sensor locally quantizes its received signal, $s_{i,t}$, and sends the quantized result, $u_{i,t}$, to the FC through an ideal channel. The quantized process follows:

(3) $$u_{i,t}=\left\{ \begin{array}{ll} 0, & \lambda_{i,t}^{\left(0\right)}<s_{i,t}<\lambda_{i,t}^{\left(1\right)}\\ 1, & \lambda_{i,t}^{\left(1\right)}\leq s_{i,t}<\lambda_{i,t}^{\left(2\right)}\\ \vdots & \vdots \\ L-1, & \;\lambda_{i,t}^{\left(L-1\right)}\leq s_{i,t}<\lambda_{i,t}^{\left(L\right)} \end{array}\right.,$$

where *L* is the quantization level and ${\left\{\lambda_{i,t}^{\left(l\right)}\right\}}_{l=0}^{L}$ are the quantization thresholds of sensor *i* at time instant *t*, specifically, $\lambda_{i,t}^{\left(0\right)}=-\infty$ and $\lambda_{i,t}^{\left(L\right)}=+\infty$. After receiving all the quantized data, $V_{t}={\left[v_{1,t},v_{2,t},\cdots ,v_{N,t}\right]}^{T}$, the FC can sequentially estimate the target state, $\theta_{t}$, using a sampling importance resampling (SIR) method [20].

However, in a non-secure environment, the sensor nodes may be subjected to various attacks. This paper considers the Byzantine attacks, in which the attackers deteriorate the system performance by capturing several nodes and forcing them to transmit false information. In the following, the attacked and un-attacked nodes are called Byzantine nodes and honest nodes, respectively. Here we assume that the attackers cannot interfere with the acquisition of the analog data, $s_{i,t}$, and the transmission of the quantized data, $u_{i,t}$. It can only locally access and modify the quantized data of Byzantine nodes. More specifically, when the sensor *i* is honest, its quantized data, $u_{i,t}$, remains unchanged. When the sensor *i* is attacked, its quantized data, $u_{i,t}=l$, can be modified to $u_{i,t}=m$ with a probability $q_{l,m}^{\left(t\right)}$ (*l*, *m* ∈ [0, *L* − 1]). Note that, the Byzantine attack parameter satisfies

(4) $$\sum_{m=0}^{L-1}q_{l,m}^{\left(t\right)}=1.$$

For the sake of compactness, we arrange the attack parameters at time instant *t* + 1 as an unknown vector, as follows:

(5) $$q_{t+1}={\left[q_{0,0}^{\left(t+1\right)},\cdots ,q_{0,L-1}^{\left(t+1\right)},q_{1,0}^{\left(t+1\right)},\cdots ,q_{1,L-1}^{\left(t+1\right)},\cdots ,q_{L-1,0}^{\left(t+1\right)},\cdots ,q_{L-1,L-1}^{\left(t+1\right)}\right]}^{T}.$$

## 3. Analysis of Attack Strategies for the Byzantine Nodes

### 3.1. Performance Metric

In order to quantify the impact of Byzantine attacks on the system performance, we set the PCRLB as the performance metric. When the attack vectors are considered, the system state model can be reformulated as follows:

(6) $$\left\{ \begin{array}{l} \theta_{t}=\tilde{F}\theta_{t-1}+w_{t}\\ q_{t}=q_{t-1}+\beta_{t} \end{array}\right.,$$

where $\beta_{t}$ is the process noise, which is assumed to be white and Gaussian with zero mean and a covariance matrix, $Q_{2}$.

In the above model, the total unknown vector is $\Theta_{t}={\left[\theta_{t}^{T},q_{t}^{T}\right]}^{T}$. Let ${\hat{\Theta }}_{t}\left(V_{1:t}\right)$ be an estimator of $\Theta_{t}$ using the observations $V_{1:t}=\left\{V_{1},\cdots ,V_{t}\right\}$ up to time instant *t*, then the mean square error matrix of the estimation error satisfies the following:

(7) $$E\left[\left({\hat{\Theta }}_{t}\left(V_{1:t}\right)-\Theta_{t}\right){\left({\hat{\Theta }}_{t}\left(V_{1:t}\right)-\Theta_{t}\right)}^{T}\right]\geq J_{t}^{-1},$$

where $J_{t}$ is the Fisher information matrix (FIM). Reference [21] shows that $J_{t}$ can be sequentially calculated through the following method:

(8) $$J_{t+1}=D_{t}^{22}-D_{t}^{21}{\left(J_{t}+D_{t}^{11}\right)}^{-1}D_{t}^{12},$$

where

(9) $$\left\{ \begin{array}{l} D_{t}^{11}=E\left\{-\nabla_{\Theta_{t}}\nabla_{\Theta_{t}}^{T}\log p\left(\Theta_{t+1}|\Theta_{t}\right)\right\},\\ D_{t}^{12}=E\left\{-\nabla_{\Theta_{t}}\nabla_{\Theta_{t+1}}^{T}\log p\left(\Theta_{t+1}|\Theta_{t}\right)\right\}={\left(D_{t}^{21}\right)}^{T} \end{array}\right.,$$

(10) $$\left\{ \begin{array}{l} D_{t}^{22}=D_{t}^{22,a}+D_{t}^{22,b}\\ D_{t}^{22,a}=E\left\{-\nabla_{\Theta_{t+1}}\nabla_{\Theta_{t+1}}^{T}\log p\left(\Theta_{t+1}|\Theta_{t}\right)\right\}\\ D_{t}^{22,b}=E\left\{-\nabla_{\Theta_{t+1}}\nabla_{\Theta_{t+1}}^{T}\log p\left(V_{t+1}|\Theta_{t+1}\right)\right\} \end{array}\right..$$

Note that the above expectations are taken with respect to the joint probability distribution, $p(\Theta_{0:t+1},V_{1:t+1})$. In our framework, the log-likelihood function, $\log p\left(V_{t+1}|\Theta_{t+1}\right)$, evaluated at $V_{t+1}=r_{t+1}$, can be expressed as follows:

(11) $$\log p\left(V_{t+1}|\Theta_{t+1}\right)=\sum_{i=1}^{N}\sum_{m=0}^{L-1}\delta \left(r_{i,t+1}-m\right)\log p_{i,t+1}^{\left(m\right)},$$

where the *δ*-function is defined as follows:

(12) $$\delta (x)=\left\{ \begin{array}{l} 1,\;x=0\\ 0,\;x\neq 0 \end{array}\right..$$

The probability, $p_{i,t+1}^{\left(m\right)}$, is

(13) $$\begin{array}{l} p_{i,t+1}^{\left(m\right)}≜\mathrm{Pr}(v_{i,t+1}=m|q_{t},\theta_{t+1})\\ =\left(1-\rho \right)\mathrm{Pr}\left(v_{i,t+1}=m|\theta_{t+1},i=Honest\right)+\rho \mathrm{Pr}\left(v_{i,t+1}=m|\theta_{t+1},i=Byzantine\right)\\ =\left(1-\rho \right)\mathrm{Pr}\left(u_{i,t+1}=m|\theta_{t+1}\right)+\rho \sum_{l=0}^{L-1}q_{l,m}^{\left(t+1\right)}\mathrm{Pr}\left(u_{i,t+1}=l|\theta_{t+1}\right) \end{array}$$

where parameter, ρ, represents the probability that any node is attacked. According to the quantization process, the conditional probability, $\mathrm{Pr}\left(u_{i,t+1}=m|\theta_{t+1}\right)$, can be calculated as follows:

(14) $$\begin{array}{l} w_{i,t+1}^{\left(m\right)}≜\mathrm{Pr}\left(u_{i,t+1}=m|\theta_{t+1}\right)=\mathrm{Pr}\left(\lambda_{i,t+1}^{\left(m\right)}\leq s_{i,t+1}<\lambda_{i,t+1}^{\left(m+1\right)}|\theta_{t+1}\right)\\ =\Psi \left(\left(\lambda_{i,t+1}^{\left(m\right)}-a_{i,t+1}\right)/\sigma \right)-\Psi \left(\left(\lambda_{i,t+1}^{\left(m+1\right)}-a_{i,t+1}\right)/\sigma \right) \end{array}$$

where Ψ(x) is the complementary cumulative distribution function of the standard normal distribution.

Based on (11), (9) and (10) can be simplified as follows:

(15) $$D_{t}^{11}=F^{T}Q^{-1}F,D_{t}^{12}=-F^{T}Q^{-1},D_{t}^{22,a}=Q^{-1},$$

(16) $$D_{t}^{22,b}=E\left\{\sum_{i=1}^{N}\sum_{m=0}^{L-1}\frac{1}{p_{i,t+1}^{\left(m\right)}}\left[\frac{\partial p_{i,t+1}^{\left(m\right)}}{\partial \Theta_{t+1}}\right]{\left[\frac{\partial p_{i,t+1}^{\left(m\right)}}{\partial \Theta_{t+1}}\right]}^{T}\right\},$$

where

(17) $$F=\left[\begin{array}{cc} \tilde{F} & 0\\ 0 & I \end{array}\right],Q=\left[\begin{array}{cc} Q_{1} & 0\\ 0 & Q_{2} \end{array}\right].$$

Note that the expectations in Equation (16) are taken with respect to $p\left(\Theta_{0:t},V_{1:t}\right)p\left(\Theta_{t+1}|\Theta_{t}\right)$.

### 3.2. Optimal Byzantine Attacks

For the attackers, the goal is to cause as much damage to the system as possible. Here, we call the event of causing maximum damage as blinding the FC, which refers to making the observations from sensors non-informative to the FC. When the Byzantine nodes adopt an attack strategy such that the observation data, $V_{t+1}$, does not contain any information about $\Theta_{t+1}$, then the Fisher information of $\Theta_{t+1}$ obtained from $V_{t+1}$ become zero, and the only beneficial way to estimate $\Theta_{t+1}$ is to use the prior information of the unknowns. From (9) and (10), we know that this is the minimum increment of Fisher information that FC can obtain at time unit *t* + 1. In other words, such an attack strategy achieves the maximum degradation to $J_{t+1}$. Based on this, the following definition is given:

**Definition** **1.** *Consider a distributed estimation framework where the parameter of interest is* $\Theta_{t+1}$ *and the contaminated observation data is* $V_{t+1}$ *. A Byzantine attack is said to be optimal if it makes the Fisher information of* $\Theta_{t+1}$ *obtained from* $V_{t+1}$ *become zero.*

**Theorem** **1.** *If a Byzantine attack is such that for any t ≥ 0,*

(18) $$D_{t}^{22,b}=0,$$

*then the observation data,*$V_{t+1}$*, does not contain any information about*$\Theta_{t+1}$.

**Proof.**  By substituting Equation (15) into Equation (8), we have the following:

(19) $$J_{t+1}=D_{t}^{22,b}+{\left(Q+FJ_{t}F^{T}\right)}^{-1}.$$

As can be seen from the equation, at time instant *t* + 1, $D_{t}^{22,b}$ is the only matrix that is related to $V_{t+1}$ and can contribute to $J_{t+1}$. Thus, to make the observations $V_{t+1}$ do not contain any information about $\Theta_{t+1}$ the attackers need to ensure that any *t* ≥ 0, $D_{t}^{22,b}=0$ □

**Proposition** **1.** *If the attack parameters satisfy that for any t ≥ 0, l, m ∈ [0, L − 1],*

(20) $$q_{l,m}^{\left(t+1\right)}=\left\{ \begin{array}{ll} 1-\frac{1}{\rho }+\frac{1}{\rho L}, & l=m\\ \frac{1}{\rho L}, & l\neq m \end{array}\right.,$$

*then the optimal Byzantine attack is achieved.*

**Proof.**  From Equation (16), we know that $D_{t}^{22,b}$ can be divided into four blocks as follows:

(21) $$D_{t}^{22,b}=\left[\begin{array}{cc} B_{11} & B_{12}\\ B_{21} & B_{22} \end{array}\right],$$

where

(22) $$B_{11}=E\left\{\sum_{i=1}^{N}\sum_{m=0}^{L-1}\frac{1}{p_{i,t+1}^{\left(m\right)}}\left[\frac{\partial p_{i,t+1}^{\left(m\right)}}{\partial \theta_{t+1}}\right]{\left[\frac{\partial p_{i,t+1}^{\left(m\right)}}{\partial \theta_{t+1}}\right]}^{T}\right\},$$

(23) $$B_{12}=E\left\{\sum_{i=1}^{N}\sum_{m=0}^{L-1}\frac{1}{p_{i,t+1}^{\left(m\right)}}\left[\frac{\partial p_{i,t+1}^{\left(m\right)}}{\partial \theta_{t+1}}\right]{\left[\frac{\partial p_{i,t+1}^{\left(m\right)}}{\partial q_{t+1}}\right]}^{T}\right\}={\left(B_{21}\right)}^{T},$$

(24) $$B_{22}=E\left\{\sum_{i=1}^{N}\sum_{m=0}^{L-1}\frac{1}{p_{i,t+1}^{\left(m\right)}}\left[\frac{\partial p_{i,t+1}^{\left(m\right)}}{\partial q_{t+1}}\right]{\left[\frac{\partial p_{i,t+1}^{\left(m\right)}}{\partial q_{t+1}}\right]}^{T}\right\}.$$

According to the definition of $p_{i,t+1}^{\left(m\right)}$, we have the following:

(25) $$\begin{array}{l} p_{i,t+1}^{\left(m\right)}=(1-\rho )\cdot w_{i,t+1}^{\left(m\right)}+\rho \sum_{l=0}^{L-1}q_{l,m}^{\left(t+1\right)}\cdot w_{i,t+1}^{\left(l\right)}\\ =(1-\rho +\rho q_{m,m}^{\left(t+1\right)})+\sum_{l\neq m}[\rho q_{l,m}^{\left(t+1\right)}-(1-\rho +\rho q_{m,m}^{\left(t+1\right)})]w_{i,t+1}^{\left(l\right)} \end{array}$$

(26) $$\frac{\partial p_{i,t+1}^{\left(m\right)}}{\partial \theta_{t+1}}=\frac{\Gamma_{i,t+1}^{m}}{\sigma \sqrt{2\pi }}\frac{\partial a_{i,t+1}}{\partial \theta_{t+1}}.$$

In the above equation, $\Gamma_{i,t+1}^{m}$ is as follows:

(27) $$\Gamma_{i,t+1}^{m}≜\left(1-\rho \right)\cdot \gamma_{i,t+1}^{m}+\rho \sum_{l=0}^{L-1}q_{l,m}^{\left(t+1\right)}\cdot \gamma_{i,t+1}^{l}=\sum_{l\neq m}\left[\rho q_{l,m}^{\left(t+1\right)}-\left(1-\rho +\rho q_{m,m}^{\left(t+1\right)}\right)\right]\gamma_{i,t+1}^{l},$$

where $\gamma_{i,t+1}^{l}≜\exp\{-{\left(\lambda_{i,t+1}^{\left(l\right)}-a_{i,t+1}\right)}^{2}/2\sigma^{2}\}-\exp\{-{\left(\lambda_{i,t+1}^{\left(l+1\right)}-a_{i,t+1}\right)}^{2}/2\sigma^{2}\}$ and it satisfies that $\sum_{l=0}^{L-1}\gamma_{i,t+1}^{l}=0$. When the attack parameters satisfy Equation (20), it can be shown that for any m∈ [0,L−1] as follows:

(28) $$\Gamma_{i,t+1}^{m}=0,$$

(29) $$p_{i,t+1}^{\left(m\right)}=1/L.$$

As a result, for any *t* ≥ 0, $B_{11}=0$, $B_{12}=0$, and $B_{22}=0$. By Theorem 1, it can be concluded that under the conditions of Equation (20), the attackers realize the optimal Byzantine attack. □

Equation (29) demonstrates that when the attack parameters satisfy Equation (20), the conditional probability, $p_{i,t+1}^{\left(m\right)}$, of any node at any time is independent of the observation data, $v_{i,t+1}$, and its value is only determined by parameter *L*. In other words, there is no information about $\Theta_{t+1}$ in the new observations. At this point, the only beneficial information that can be utilized is the prior information of the unknowns.

By noticing that $q_{m,m}^{\left(t+1\right)}\geq 0$, we obtain the following:

(30) ρ≥(L−1)/L.

In general, the stronger the attackers are, the larger the value of parameter ρ will be. In order to achieve optimal Byzantine attacks and minimize the requirements on the attackers’ capabilities, it is desirable to set $\rho =\rho_{\min}≜\left(L-1\right)/L$. At this point, the optimal attack parameters become the following:

(31) $$q_{l,m}^{\left(t+1\right)}=\left\{ \begin{array}{ll} 0, & l=m\\ 1/\left(L-1\right), & l\neq m \end{array}\right..$$

When *L* = 2 and $\rho =\rho_{\min}=1/2$, the attack parameters in Equation (31) become $q_{0,0}^{\left(t+1\right)}=q_{1,1}^{\left(t+1\right)}=0$ and $q_{0,1}^{\left(t+1\right)}=q_{1,0}^{\left(t+1\right)}=1$, which implies that to achieve the optimal Byzantine attack, all Byzantine nodes must flip their own local quantized measurements with a probability of ‘1’.

**Figure 2:**
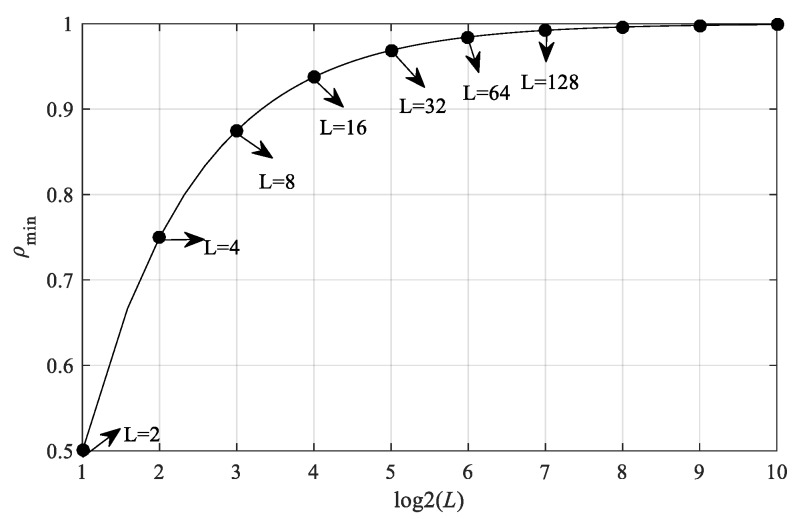
The relationship between $\rho_{\min}$ and *L*.

Figure 2 depicts the relationship between $\rho_{\min}$ and *L*. It can be observed that when *L* gradually increases, $\rho_{\min}$ also increases. If $\rho_{\min}$ is considered as the proportion of attacked nodes in the network, when *L* = 2, $\rho_{\min}$= 0.5, it means that in order to achieve optimal Byzantine attacks, the attackers need to capture at least 50% of sensor nodes in the network. As *L* increases, the number of nodes that need to be captured also increases, which places a higher requirement for the attackers. In the extreme cases (i.e., *L* → ∞, $\rho_{\min}$= 1), all nodes in the network must be captured by the attackers.

### 3.3. Sub-Optimal Byzantine Attacks

In the analysis of optimal Byzantine attacks, it is assumed that the FC knows the probability that each node is captured (i.e., *ρ*), but it is not clear about the real attribute of each node (i.e., malicious or honest.). In that case, it is possible for attackers to make the new observations of all nodes containing no information about $\Theta_{t+1}$. Recently, the work in References [2,18] shows that, for some classes of Byzantine attacks with a sufficient number of observations, the FC is able to perfectly identify and categorize the attacked sensors into different groups. Thus, in this section, we further derive the most destructive Byzantine attack strategy when the FC knows the real attributes of all nodes. It is worth mentioning that under this case, the least amount of Fisher information that can be obtained to develop the PCRLB is the information contained in the observations from un-attacked sensors. In other words, if the data contribution from each attacked sensor observation to the FIM becomes zero, then the maximum degradation of the PCRLB can be achieved. Based on this, the following definition is given.

**Definition** **2.** *Consider a distributed estimation framework where the FC knows the attribute of each node. A Byzantine attack is said to be suboptimal if it makes the Fisher information of $\Theta_{t+1}$ obtained from each attacked sensor observation become zero.*

When the true states of all nodes are known to the FC, the log-likelihood function of received data can be expressed as follows:

(32) $$\log p\left(V_{t+1}|q_{t+1},\theta_{t+1}\right)=\sum_{i\in S_{0}}\sum_{m=0}^{L-1}\delta \left(r_{i,t+1}-m\right)\log p_{i\in S_{0},t+1}^{\left(m\right)}+\sum_{i\in S_{1}}\sum_{m=0}^{L-1}\delta \left(r_{i,t+1}-m\right)\log p_{i\in S_{1},t+1}^{\left(m\right)},$$

where $S_{0}$ and $S_{1}$ are the sets of honest sensors and Byzantine sensors, respectively. The probabilities $p_{i\in S_{0},t+1}^{\left(m\right)}$ and $p_{i\in S_{1},t+1}^{\left(m\right)}$ are defined as follows:

(33) $$\left\{ \begin{array}{l} p_{i\in S_{0},t+1}^{\left(m\right)}≜\mathrm{Pr}\left(v_{i,t+1}=m|\theta_{t+1},i\in S_{0}\right)=w_{i,t+1}^{\left(m\right)}\\ p_{i\in S_{1},t+1}^{\left(m\right)}≜\mathrm{Pr}\left(v_{i,t+1}=m|q_{t},\theta_{t+1},i\in S_{1}\right)=\sum_{l=0}^{L-1}q_{l,m}^{\left(t+1\right)}w_{i,t+1}^{\left(l\right)} \end{array}\right.,$$

and they satisfy that $\sum_{m}p_{i\in S_{0},t+1}^{\left(m\right)}=\sum_{m}p_{i\in S_{1},t+1}^{\left(m\right)}=1$. By substituting Equation (32) into Equation (10), we get the following:

(34) $$D_{t}^{22,b}=H_{t}^{22,0}+\sum_{i\in S_{1}}H_{i,t}^{22,1},$$

where the matrices $H_{t}^{22,0}$ and $H_{i,t}^{22,1}$ are defined as follows:

(35) $$H_{t}^{22,0}=E\left\{{\sum_{i\in S_{0}}\sum_{m=0}^{L-1}\frac{1}{p_{i\in S_{0},t+1}^{\left(m\right)}}\left[\frac{\partial p_{i\in S_{0},t+1}^{\left(m\right)}}{\partial \Theta_{t+1}}\right]\left[\frac{\partial p_{i\in S_{0},t+1}^{\left(m\right)}}{\partial \Theta_{t+1}}\right]}^{T}\right\},$$

(36) $$H_{i,t}^{22,1}≜E\left\{\sum_{m=0}^{L-1}\frac{1}{p_{i\in S_{1},t+1}^{\left(m\right)}}\left[\frac{\partial p_{i\in S_{1},t+1}^{\left(m\right)}}{\partial \Theta_{t+1}}\right]{\left[\frac{\partial p_{i\in S_{1},t+1}^{\left(m\right)}}{\partial \Theta_{t+1}}\right]}^{T}\right\}.$$

**Theorem** **2.** *If the Byzantine attacks are such that for any*$i\in S_{1}$*and*t≥0,

(37) $$H_{i,t}^{22,1}=0,$$

*then the observation data of each attacked node does not contain any information about*$\Theta_{t+1}$.

**Proof.**  By substituting Equation (34) into Equation (8), we get the following:

(38) $$J_{t+1}={\left(Q+FJ_{t}F^{T}\right)}^{-1}+H_{t}^{22,0}+\sum_{i\in S_{1}}H_{i,t}^{22,1}.$$

As can be seen from Equation (38), at time instant *t* + 1, $\sum_{i\in S_{1}}H_{i,t}^{22,1}$ is the only matrix that is related to the Byzantine nodes’ observations and can contribute to $J_{t+1}$. Thus, to make each attacked sensor observation containing no information about $\Theta_{t+1}$, the attackers need to ensure that $H_{i,t}^{22,1}=0$ for any *t* ≥ 0 and $i\in S_{1}$. □

**Proposition** **2.** *Given the Equations (32) and (34), if the Byzantine attack parameters satisfy that for any t ≥ 0, l,*m∈ [0,L−1],

(39) $$q_{l,m}^{\left(t+1\right)}=q_{m,m}^{\left(t+1\right)}=1/L,$$

*then the suboptimal Byzantine attack is achieved.*

**Proof.**  Since $\Theta_{t}={\left[\theta_{t}^{T},q_{t}^{T}\right]}^{T}$, $H_{i,t}^{22,1}$ can also be further divided into four bocks as follows:

$$H_{i,t}^{22,1}=\left[\begin{array}{cc} H_{i,11} & H_{i,12}\\ H_{i,21} & H_{i,22} \end{array}\right],$$

where

(40) $$\begin{array}{l} H_{i,11}=E\left\{{\sum_{m=0}^{L-1}\frac{1}{p_{i\in S_{1},t+1}^{\left(m\right)}}\left[\frac{\partial p_{i\in S_{1},t+1}^{\left(m\right)}}{\partial \theta_{t+1}}\right]\left[\frac{\partial p_{i\in S_{1},t+1}^{\left(m\right)}}{\partial \theta_{t+1}}\right]}^{T}\right\}\\ H_{i,12}=E\left\{{\sum_{m=0}^{L-1}\frac{1}{p_{i\in S_{1},t+1}^{\left(m\right)}}\left[\frac{\partial p_{i\in S_{1},t+1}^{\left(m\right)}}{\partial \theta_{t+1}}\right]\left[\frac{\partial p_{i\in S_{1},t+1}^{\left(m\right)}}{\partial q_{t+1}}\right]}^{T}\right\}={\left(H_{i,21}\right)}^{T}\\ H_{i,22}=E\left\{{\sum_{m=0}^{L-1}\frac{1}{p_{i\in S_{1},t+1}^{\left(m\right)}}\left[\frac{\partial p_{i\in S_{1},t+1}^{\left(m\right)}}{\partial q_{t+1}}\right]\left[\frac{\partial p_{i\in S_{1},t+1}^{\left(m\right)}}{\partial q_{t+1}}\right]}^{T}\right\} \end{array}$$

From (33), we know the following:

(41) $$\frac{\partial p_{i\in S_{1},t+1}^{\left(m\right)}}{\partial \theta_{t+1}}=\frac{{Χ}_{i\in S_{1},t+1}^{m}}{\sigma \sqrt{2\pi }}\frac{\partial a_{i,t+1}}{\partial \theta_{t+1}},$$

where parameter ${Χ}_{i\in S_{1},t+1}^{m}≜\sum_{l=0}^{L-1}q_{l,m}^{\left(t+1\right)}\cdot \gamma_{i,t+1}^{l}$. When the condition Equation (39) is satisfied,

(42) $${Χ}_{i\in S_{1},t+1}^{m}=q_{m,m}^{\left(t+1\right)}\cdot \gamma_{i,t+1}^{m}+\sum_{l\neq m}^{L-1}q_{l,m}^{\left(t+1\right)}\cdot \gamma_{i,t+1}^{l}=\sum_{l\neq m}^{L-1}(q_{l,m}^{\left(t+1\right)}-q_{m,m}^{\left(t+1\right)})\gamma_{i,t+1}^{l}=0,$$

(43) $$p_{i\in S_{1},t+1}^{\left(m\right)}=q_{m,m}^{\left(t+1\right)}\sum_{l=0}^{L-1}w_{i,t+1}^{\left(l\right)}=q_{m,m}^{\left(t+1\right)}=1/L.$$

As a result, for any $i\in S_{1}$ and *t* ≥ 0, $H_{i,t}^{22,1}=0$. By Theorem 2, it can be concluded that under the conditions of Equation (39), the suboptimal Byzantine attacks are achieved. □

From Equation (43), we know that when the attack parameters satisfy Equation (39), the conditional probability, $p_{i\in S_{1},t+1}^{\left(m\right)}$, becomes independent of the attacked sensor observations. In other words, the attacked sensor observations received by the FC do not contain any valid information about the unknowns. Therefore, the FC can only use the prior information and the un-attacked sensor observations to estimate the unknowns.

### 3.4. Numerical Results

In this subsection, we present numerical results in support of our analysis on Byzantine attacks in a target tracking problem. It is assumed that the mobile target is free to move within a 600 × 600 square area. The target motion model is assumed to be a near constant velocity model and the state transition matrix and the covariance matrix of the process noise are defined as follows:

(44) $$\tilde{F}=\left[\begin{array}{cccc} 1 & 0 & T & 0\\ 0 & 1 & 0 & T\\ 0 & 0 & 1 & 0\\ 0 & 0 & 0 & 1 \end{array}\right],\;Q_{1}=q\left[\begin{array}{cccc} T^{3}/3 & 0 & T^{2}/2 & 0\\ 0 & T^{3}/3 & 0 & T^{2}/2\\ T^{2}/2 & 0 & T & 0\\ 0 & T^{2}/2 & 0 & T \end{array}\right],$$

where *T* is the observation interval and *q* is a process noise parameter. In the monitoring area, *N* sensor nodes are evenly distributed and the total number of Byzantine nodes is M=ρ·N. The observations between nodes are assumed to be independent. The total observation time is $T_{s}$. All nodes adopt the same entropy-based heuristic quantization scheme proposed in Reference [22] at any time and all Byzantine nodes modify their local quantized observations according to the settings of Equation (20). The default parameter settings are listed in Table 1.

**Figure 3:**
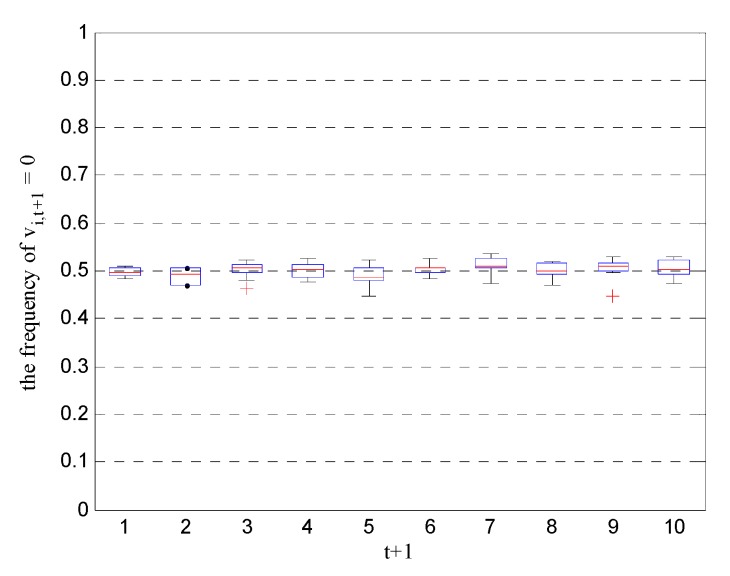
Frequency of $v_{i,t+1}=0$ for all nodes and all time units under optimal Byzantine attacks.

In simulations, we calculated the frequency of $v_{i,t+1}=0$ for all nodes and all time units over 1000 randomized trials. The results listed in Figure 3 show that when the Byzantine attack parameters satisfy Equation (20), the frequency of $v_{i,t+1}=0$ is approximately 0.5 for all nodes and all time units, resulting in equiprobable quantized values at the FC. In other words, the conditional probabilities of the received data become $p_{i,t+1}^{\left(0\right)}\approx p_{i,t+1}^{\left(1\right)}\approx 1/2$, which is consistent with the theoretical results of Equation (29) under optimal Byzantine attacks.

**Figure 4:**
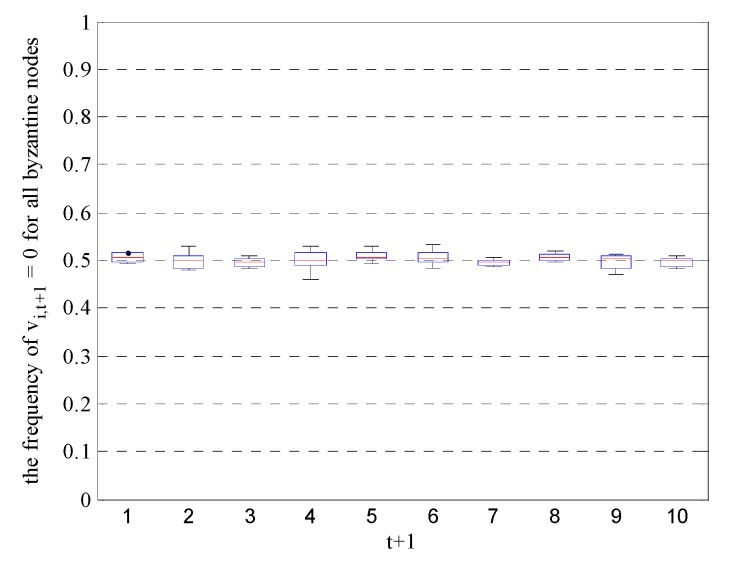
Frequency of $v_{i,t+1}=0$ for all Byzantine nodes and all time units under suboptimal Byzantine attacks.

Next, we assume that the first M sensor nodes are Byzantine nodes and their attack parameters follow Equation (39). However, these malicious nodes are correctly identified by the FC. Under this circumstance, we also calculated the frequency of $v_{i,t+1}=0$ for all Byzantine nodes and all time units over 1000 randomized trials. The results, listed in Figure 4, show that when the Byzantine parameters follow Equation (39), the frequency of $v_{i,t+1}=0$ is approximately 0.5 for all Byzantine nodes and all time units. In other words, the conditional probabilities of received data become $p_{i\in S_{1},t+1}^{\left(0\right)}\approx p_{i\in S_{1},t+1}^{\left(1\right)}\approx 1/2$, which is consistent with the result of Equation (43) under suboptimal Byzantine attacks.

## 4. Identification of Byzantine Nodes

### 4.1. The Modified SIR Filter

In order to alleviate the negative impact of Byzantine attacks on the system performance, a modified SIR filter is proposed. Table 2 shows the main flow of the filter.

In the above filter, $N_{p}$ is the total number of valid samples, $p(\theta_{0})$ is the initial distribution of the target state, and $p(\theta_{t}|\theta_{t-1})$ denotes the particle prediction function. In the resampling step, the number of copies of the particle $\theta_{t}^{\left(i\right)}$ is proportional to its weight, $\tau_{t}^{\left(i\right)}$. In step 7, we adopt the Byzantine identification scheme proposed in [23] to determine the attributes of sensor nodes. However, the scheme in [23] only considers binary quantization and is not completely suitable for our cases. So, some small modifications are made here. First, parameter ${\hat{\gamma }}_{i,t}$ is calculated through the following formula:

(45) $${\hat{\gamma }}_{i,t}=\left(t{\hat{\gamma }}_{i,t-1}+\chi \right)/t,$$

where χ is defined as follows:

(46) $$\chi =\left\{ \begin{array}{l} 0,\;v_{i,t}={\hat{v}}_{i,t}\\ 1,\;v_{i,t}\neq {\hat{v}}_{i,t} \end{array}\right..$$

In the above equations, ${\hat{v}}_{i,t}$ is the observation estimated based on ${\tilde{\theta }}_{t}$ and ${\hat{\gamma }}_{i,t}$ characterizes the probability that node *i* modifies the quantized observation at time *t*, and its value is related to the historical observations $\left\{v_{i,1},\cdots ,v_{i,t}\right\}$ and the preliminary target state estimation ${\tilde{\theta }}_{t}$. From Equation (31), we know that in order to achieve the optimal Byzantine attacks with minimal cost, the Byzantine nodes must modify the original quantized data to other possible values. Thus, we use the following statistic to determine the nodes’ states:

(47) $$\Lambda_{i,t}=\left|\frac{{\hat{\gamma }}_{i,t}-0}{{\hat{\gamma }}_{i,t}-1}\right|.$$

In the above equation, the numerator $\Lambda_{i,t}$ describes the deviation between ${\hat{\gamma }}_{i,t}$ and the probability that honest nodes modify the quantized observations. The denominator describes the deviation between ${\hat{\gamma }}_{i,t}$ and 1 (i.e., the probability that the malicious nodes modify the quantized observations under optimal Byzantine attacks). When $\Lambda_{i,t}>1$, the denominator is smaller than the numerator. Thus, we incline to accept that node *i* is a Byzantine node. Otherwise, node *i* is identified as an honest node.

After determining the states of all nodes, we prune out the observation data from all Byzantine nodes and use the remaining observations to update the particle set and output the final estimate of the unknown target state.

### 4.2. Numerical Results

In this subsection, the performance of the modified SIR algorithm is evaluated. For the mobile target, it is assumed to freely move in a 600 × 600 square area. The real initial target state is $\theta_{0}={\left[5,5,6,6\right]}^{T}$. The state dynamics are modeled using the matrix $\tilde{F}$ and $Q_{1}$, defined in Equation (44). There are *N* = 100 evenly distributed sensors in the monitoring area and the total number of Byzantine nodes is *M* = 40. The total observation time is $T_{s}$= 80. All nodes adopt the same quantization scheme as in Section 3.4. The Byzantine attack parameters follow the Equation (31). For the filter, the initial state particles are generated from $p(\theta_{0})$, which is assumed to be Gaussian, and its expectation and variance are $E(\theta_{0})$ and $Var(\theta_{0})$, respectively. The default parameter settings are listed in Table 3.

**Figure 5:**
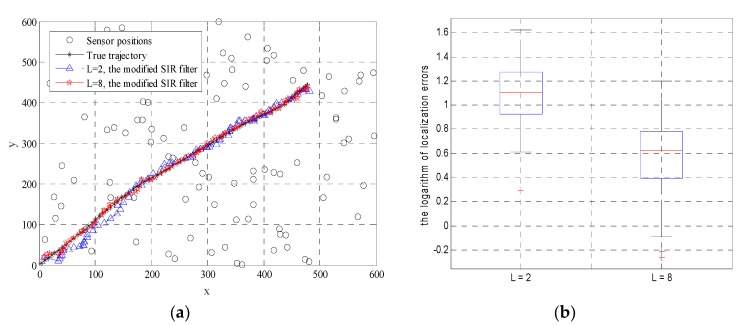
Estimation results of the modified SIR filter: (**a**) The estimated tracks of the moving target when *L* = 2 and *L* = 8; and (**b**) the logarithm of the localization errors.

**Figure 6:**
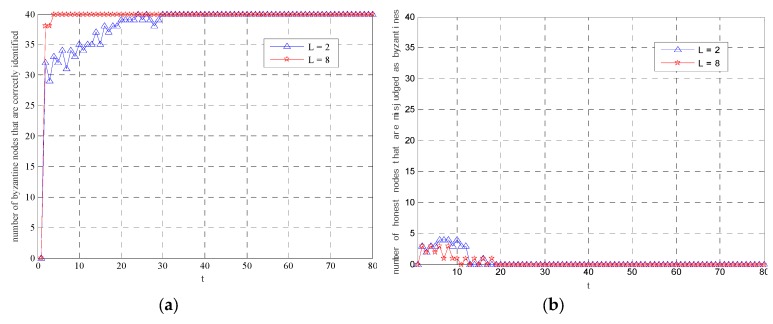
The detection rate and the false detection rate of the modified SIR filter: (**a**) The number of Byzantine nodes that are correctly identified; (**b**) the number of honest nodes that are misjudged as Byzantines.

Figure 5 shows the estimation results in a particular realization. From Figure 5a, it can be observed that by employing the Byzantine identification scheme, the estimated tracks can be close to the true trajectory of the moving target. Figure 5b shows the improvement of tracking performance in the sense of localization errors when *L* increases. In our paper, the localization errors are defined as the distances between the estimated coordinates and the true locations of the moving target. It can be observed that the median of localization errors when *L* = 2 is 3 times larger than that of localization errors when *L* = 8. Figure 6 shows the detection rate and false detection rate in this realization. It can be seen that the modified SIR filter can identify all the Byzantine nodes within a certain period of time. More precisely, when *L* = 2, all the Byzantine nodes are detected during the first 30 rounds of tracking. When *L* = 8, the time required to identify all malicious nodes is shorter (i.e., *t* = 5). Combined with the former results in Figure 5, it can be concluded that when *L* increases, both the tracking results and the security performance of the system are improved.

## 5. Conclusions

In summary, the problem of target tracking with quantized sensor observations is considered in the presence of Byzantine attacks. From the perspective of attackers, we have analyzed the most destructive effect of Byzantine attacks on the system performance in the sense of PCRLB. The results showed that the fusion center becomes ‘blind’ to the information from all sensors when the Byzantine attack parameters follow Equation (20). In such a case, the total observations received by the fusion center do not contain any information about the target parameters, which generates the maximum degradation to the PCRLB. When the Byzantine attack parameters follow Equation (39), only the attacked observations do not contain any information about the target parameters, which generates the maximum degradation to the PCRLB when all the Byzantine nodes are correctly identified by the fusion center. We have also proposed a modified SIR filter to minimize the negative impact of attackers on the system. Results show that increasing the quantization level can effectively improve the estimation performance and security performance of the system.

## Figures and Tables

**Table 1:** Default parameter settings for Byzantine attacks.

Parameters	Values
Network size	600 × 600
*N*	10
$T_{s}$	10
*T*	1
*q*	0.16
$P_{0}$	25,000
*L*	2
ρ	0.5
σ	0.1

**Table 2:** The main flow of modified SIR filter.

**The Modified SIR Filter**	
1	Initialization: Set *t* = 1, randomly draw $N_{p}$ particles $\theta_{0}^{\left(i\right)}$ from $p\left(\theta_{0}\right)$ and set $w_{0}^{\left(i\right)}=1/N_{p}$.
2	While $t\leq T_{s}$ do
3	Prediction: $\theta_{t}^{\left(i\right)}\sim p(\theta_{t}|\theta_{t-1}^{\left(i\right)})$.
4	Calculating the weights: ${\tilde{\tau }}_{t}^{\left(i\right)}\propto \tau_{t-1}^{\left(i\right)}\cdot p(V_{t}|\theta_{t}^{\left(i\right)})$, $\tau_{t}^{\left(i\right)}={\tilde{\tau }}_{t}^{\left(i\right)}/\sum_{j=1}^{N_{p}}{\tilde{\tau }}_{t}^{\left(j\right)}$.
5	Resampling according to the weights: $\left\{\theta_{t}^{\left(i\right)},N_{P}^{-1}\}\sim \{\theta_{t}^{\left(i\right)},\tau_{t}^{\left(i\right)}\right\}$.
6	Preliminary estimation: ${\tilde{\theta }}_{t}=1/N_{P}\cdot \sum_{i=1}^{N_{P}}\theta_{t}^{\left(i\right)}$.
7	Byzantine node identification: Determine the states of all nodes based on ${\tilde{\theta }}_{t}$ and $V_{t}$, and prune out the attacked observations from all Byzantine nodes.
8	Update the particle set with the remaining observations and output the final target state estimation, ${\hat{\theta }}_{t}$, at time unit *t*.
9	Set *t* = *t* + 1.
10	End While

**Table 3:** Default parameter settings for modified SIR filter.

Parameters	Values
Network size	600 × 600
*N*	100
*M*	40
$T_{s}$	80
*T*	1
*q*	0.16
$P_{0}$	25,000
σ	0.1
$E(\theta_{0})$	${\left[10,20,6,6\right]}^{T}$
$Var(\theta_{0})$	diag([36,36,0.04,0.04])
$N_{p}$	100
